# Analysis and visual summarization of molecular dynamics simulation

**DOI:** 10.1186/1758-2946-6-S1-O16

**Published:** 2014-03-11

**Authors:** Fredrick Robin Devadoss, Victor Paul Raj

**Affiliations:** 1 Department of Chemistry, University of Konstanz, 78457 Konstanz, Germany

## 

Molecular dynamics (MD) simulation, a standard technique used to study the dynamical properties of biomolecules, is very useful in collecting the trajectories, a series of snapshots – the coordinates of the system - of larger systems for longer simulation times. These MD generated trajectories are huge in size (many gigabytes) and the data analysis may take much longer time than the data generation. Managing the large amount of data and presenting them in a flexible and comprehensible manner are the major challenges. Analyzing these trajectories with standard parameter like root-mean square deviation (RMSD) may not reveal the most interesting properties of the dynamics.

To overcome these challenges, C^α^ torsion angles [1] - torsion angles build by four consecutive C^α^ atoms - are highly valuable as similarity measure on a substructure scale and to find major events - the information on the time at which a transition occurs (temporal domain) and the local structural changes (spatial domain) of it is combinedly called as “event”- occurring in the course of the MD simulation. By calculating the time series of the C^α^ torsion angles and their clustering it is possible to determine the mechanistic details on a residual length scale and find major events occurring in the simulation of large proteins or protein complexes. The main advantage of the C^α^ torsion angle criterion is that it does not depend on a previous alignment of the structures, and that the direction of the change is also defined. Heat maps of C^α^ torsion angle give nice graphical representations of processes described by the MD simulations. Clustering of snapshots according to the specific C^α^ torsion angles is used to automatically find the spatial domains of the structural changes. If all the snapshots are assigned to a single cluster, then those residues are considered as rigid core and the remaining residues are considered as flexible parts. The temporal domain can be characterized in more detail by finding continuous time intervals assigned to a single cluster as (meta) stable structures and time intervals where the assignment jumps between two clusters as transitional periods. Since the outliers can be removed from the fuzzy clusters, starts and ends of time patches now qualify as important events for the underlying substructure and structural changes of larger regions are caused by an accumulation of such substructure events.

DNA polymerase I – the open ternary complex of the large fragment of Thermus aquaticus DNA polymerase I (Klentaq1), which is used here as a practical application for C^α^ torsion angle based analysis, shows a hand-like arrangement, including a thumb, a palm and a finger domain [2]. The catalytic cycle leading to nucleotide insertion comprises several steps including a large structural rearrangement in the form of a movement of the finger domain towards the thumb domain, i.e. the transition from the open to the closed form. Molecular dynamics simulation were performed using the AMBER 10 suite of programs [3]. To get the visual picture of the ongoing processes, the C^α^ torsion angles with the differences to the crystal structure of the open form were plotted as heat map. The rigid and the flexible parts were clearly seen with no or a large number of significant changes, respectively, from the heat map. Once the C^α^ torsion angles corresponding to the rigid parts are removed, the remaining regions change only in a specific time interval of the simulation. The spatial and temporal domains of the structural changes were identified automatically by clustering of snapshots (using KNIME [4]) and finding the continuous time intervals, respectively.
